# Vitamin D, Autoimmune Disease and Rheumatoid Arthritis

**DOI:** 10.1007/s00223-019-00577-2

**Published:** 2019-07-08

**Authors:** Stephanie R. Harrison, Danyang Li, Louisa E. Jeffery, Karim Raza, Martin Hewison

**Affiliations:** 1 Institute of Metabolism and Systems Research, The University of Birmingham, Birmingham, B15 2TT UK; 2 Department of Rheumatology, Sandwell and West, Birmingham Hospitals NHS Trust, Birmingham, B18 7QH UK; 3 Institute of Translation Medicine, The University of Birmingham, Birmingham, B15 2TT UK; 4 Institute of Inflammation and Ageing, Arthritis Research UK Rheumatoid Arthritis Pathogenesis Centre of Excellence and MRC Arthritis Research UK Centre for Musculoskeletal Ageing Research, University of Birmingham, Birmingham, B15 2TT UK; 5 Centre for Endocrinology, Diabetes and Metabolism, Birmingham Health Partners, Birmingham, B15 2TT UK

**Keywords:** Vitamin D, Vitamin D receptor, Autoimmune disease, Rheumatoid arthritis, Inflammation, T cell

## Abstract

Vitamin D has been reported to influence physiological systems that extend far beyond its established functions in calcium and bone homeostasis. Prominent amongst these are the potent immunomodulatory effects of the active form of vitamin D, 1,25-dihydroxyvitamin D3 (1,25-(OH)_2_D3). The nuclear vitamin D receptor (VDR) for 1,25-(OH)_2_D3 is expressed by many cells within the immune system and resulting effects include modulation of T cell phenotype to suppress pro-inflammatory Th1 and Th17 CD4+ T cells and promote tolerogenic regulatory T cells. In addition, antigen-presenting cells have been shown to express the enzyme 1α-hydroxylase that converts precursor 25-hydroxyvitamin D3 (25-OHD3) to 1,25-(OH)_2_D3, so that immune microenvironments are able to both activate and respond to vitamin D. As a consequence of this local, intracrine, system, immune responses may vary according to the availability of 25-OHD3, and vitamin D deficiency has been linked to various autoimmune disorders including rheumatoid arthritis (RA). The aim of this review is to explore the immune activities of vitamin D that impact autoimmune disease, with specific reference to RA. As well as outlining the mechanisms linking vitamin D with autoimmune disease, the review will also describe the different studies that have linked vitamin D status to RA, and the current supplementation studies that have explored the potential benefits of vitamin D for prevention or treatment of RA. The overall aim of the review is to provide a fresh perspective on the potential role of vitamin D in RA pathogenesis and treatment.

**Electronic supplementary material:**

The online version of this article (10.1007/s00223-019-00577-2) contains supplementary material, which is available to authorized users.

## Introduction

Vitamin D is a secosteroid which can be obtained from the diet (in particular from oily fish, eggs, dairy products and fortified foods). However, in humans, the majority of vitamin D is synthesised in the skin from the precursor molecule 7-dehydrocholesterol, which undergoes a series of UV light-mediated modifications to generate parental vitamin D3 [1]. Vitamin D was first recognised for its role in bone mineralisation and calcium regulation, with vitamin D deficiency associated with the bone disease rickets [2]. More recently, vitamin D has been reported to exert many extra-skeletal effects [3] with association studies linking vitamin D status to a broad range of human health issues. Prominent amongst these is the proposed role of vitamin D in the pathophysiology of autoimmune disease, including insulin-dependent type 1 diabetes mellitus (T1D) [4], autoimmune thyroid disease [5, 6], multiple sclerosis (MS), inflammatory bowel disease (IBD) [7], systemic lupus erythematosus (SLE) [8] and rheumatoid arthritis (RA) [9]. The underlying mechanisms by which vitamin D impacts autoimmune disease remain elusive, and it is still not clear whether vitamin D deficiency contributes to autoimmune disease pathogenesis or whether it is marker of disease progression and severity. In this article, we address these issues with specific reference to the role of vitamin D in RA. We discuss the potential clinical significance, and the mechanisms of action of vitamin D in RA, and suggest areas where future research is needed.

## Vitamin D in Health

Humans obtain vitamin D in two forms; vitamin D2 (ergocalciferol, derived from plant ergosterols) and vitamin D3 (cholecalciferol), which differ in the number/location of double carbon–carbon bonds. Vitamin D2 has only two C=C bonds, whilst vitamin D3 has three, affording D2 a lower affinity for vitamin D-binding protein (DBP), increasing clearance and reduced bioavailability; thus, vitamin D3 is the main form of vitamin D used by humans. Generation of active, hormonal, vitamin D3 involves a series of non-enzymatic and enzymatic processes. Firstly, 7-dehydrocholesterol is converted to vitamin D3 when exposed to UVB light in the dermis. Vitamin D3 is then converted to 25-hydroxycholecalciferol (25-OHD3) by the enzyme 25-hydroxylase (CYP2R1) located predominantly in the liver [10]. The final step in generating active 1,25-dihydroxycholecalciferol (1,25-(OH)_2_D3) is mediated by the enzyme 25-hydroxyvitamin D-1α-hydroxylase (CYP27B1), which is abundant in the proximal tubule cells of the kidney [11]. This step is responsible for the majority of circulating 1,25-(OH)_2_D3, but CYP27B1 is also expressed by a variety of non-renal tissues [12], including several immune cell subsets [13–15], suggesting that these cells also have significant capacity to generate 1,25-(OH)_2_D3, with this process being instrumental in various autocrine and paracrine cell responses to vitamin D [16, 17]. Moreover, whilst renal CYP27B1 is tightly regulated by parathyroid hormone, fibroblast growth factor 23 and 1,25-(OH)_2_D3 itself, extra-renal CYP27B1 does not appear to be subject to the same regulatory constraints [18, 19]. Instead extra-renal synthesis of 1,25-(OH)_2_D3 appears to be more dependent on the availability of 25-OHD3. In view of the fact that 25-OHD3 is the main determinant of serum vitamin D status, it is highly likely that extra-renal synthesis of 1,25-(OH)_2_D3 will therefore be strongly influenced by vitamin D deficiency/sufficiency. The availability of 25-OHD3 is also affected by circulating serum carrier proteins for vitamin D: Vitamin D-Binding Protein (VBP), and to a lesser extent albumin [20, 21]. Levels of 1,25-(OH)_2_D3 in either renal or extra-renal settings are also influenced by catabolism of 1,25-(OH)_2_D3 to inactive metabolites which is primarily controlled by the enzyme 24-hydroxylase (CYP24A1) [22], with mutations in the *CYP24A1* gene being associated with dysregulated catabolism of 1,25-(OH)_2_D3 [23].

**Fig. 1:**
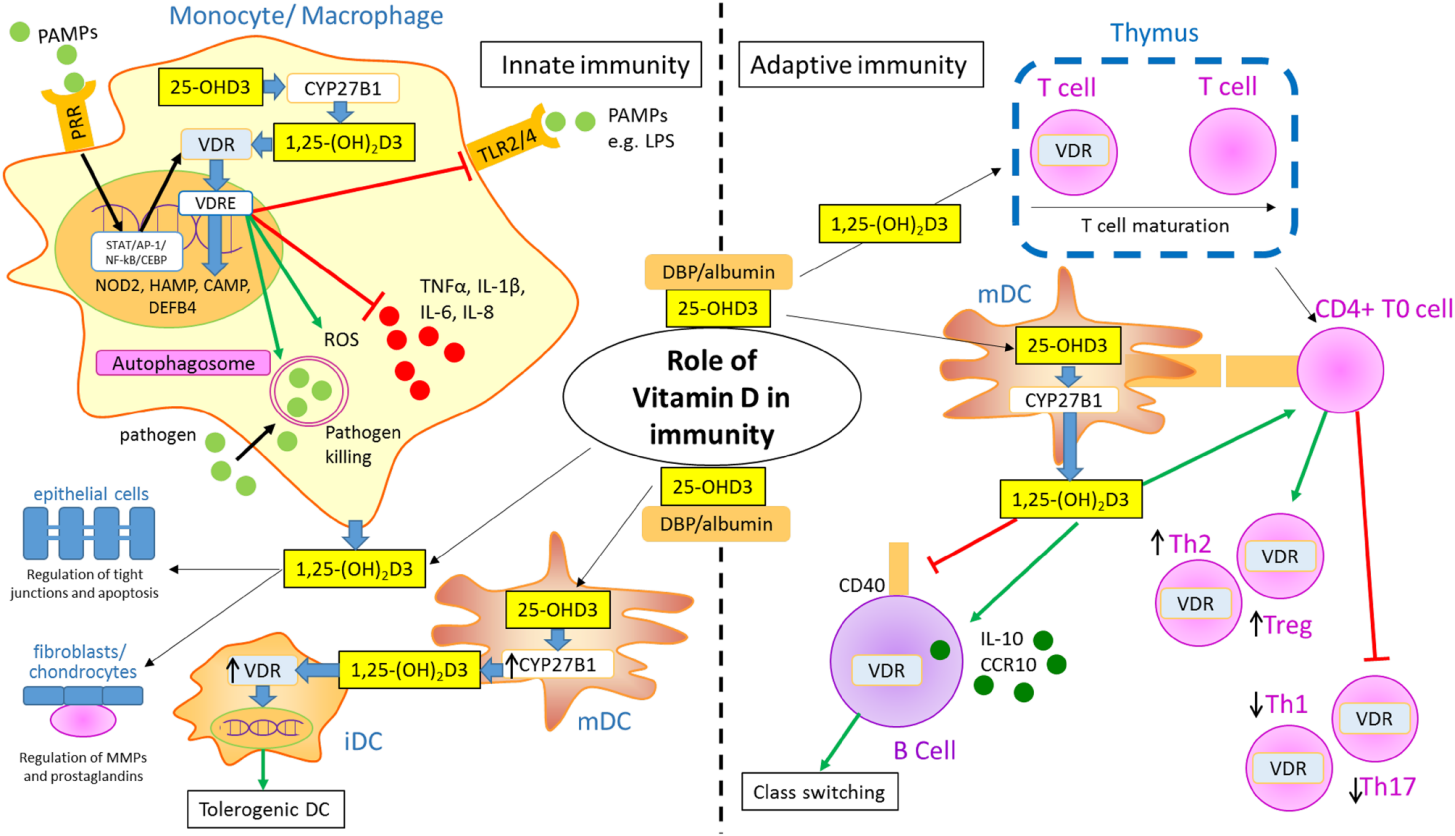
Role of vitamin D in the immune system. Schematic representation of cells from the innate and adaptive immune systems. Monocyte/macrophages from the innate immune system expression pattern recognition receptors (PRR) such as toll-like receptors (TLR), and response to pathogen-associated molecular patterns (PAMPs) such as lipopolysaccharide (LPS). PAMP–PRR responses include induction of transcription to increased expression of the vitamin D receptor (VDR) and the vitamin D-activating enzyme 1α-hydroxylase (CYP27B1) via STAT 1 or 5, AP-1, NF-κB or CEBP response elements. This increases monocyte/macrophage capacity to metabolise 25-hydroxyvitamin D3 (25-OHD3) to 1,25-dihydroxyvitamin D3 (1,25-(OH)_2_D3), which then interacts with VDR to regulate gene expression via DNA vitamin D response elements (VDRE). Prominent target genes for regulation by 1,25-(OH)_2_D3 include Nucleotide-binding oligomerization domain-containing protein 2 (NOD2), hepcidin antimicrobial protein (HAMP), cathelicidin (CAMP) and β-defensin 2 (DEFB4). 1,25-(OH)_2_D3 also enhances pathogen killing by inducing autophagy and reactive oxygen species (ROS), but acts to inhibit inflammation by suppressing inflammatory cytokines and expression of TLR2/4. Monocytes/macrophages may contribute to local levels of 1,25-(OH)_2_D3 which may then act on non-immune cells such as local tissue fibroblasts, chondrocytes or epithelial cells. For other innate immunity cells such as dendritic cells (DC), differentiation of these cells from immature (iDC) to mature (mDC) phenotypes is associated with increased expression of CYP27B1 but decreased expression of VDR, suggesting local conversion of 25-OHD3 to 1,25-(OH)2D3, which results in a paracrine effect to generate tolerogenic DC. Synthesis of 1,25-(OH)_2_D3 by mDC may also have paracrine effects on cells from the adaptive immune system such as T cells which, when activated, express VDR and respond to 1,25-(OH)_2_D3 by inducing Th2 and Treg phenotypes whilst suppressing Th1 and Th17 inflammatory phenotypes. 1,25-(OH)_2_D3 can also act on B cells to decrease CD40 expression and enhance class switching

Active 1,25-(OH)_2_D3 has an established role in regulating bone metabolism and calcium homeostasis, but it has also been shown to modulate key mechanisms in innate and adaptive immunity [24–26] (Fig. 1). The nuclear receptor for 1,25-(OH)_2_D3 (the vitamin D receptor (VDR)) is expressed by a plethora of immune cells, including monocytes/macrophage, dendritic cells (DC), neutrophils and B and T cells [27, 28]. This, coupled with the capacity for localised CYP27B1-mediated synthesis of 1,25-(OH)_2_D3 by monocytes, macrophages and DC [13, 14], supports a role for vitamin D as an important contributor to normal immune function. The remainder of the review will focus on this activity of vitamin D and the impact that this may have on the chronic inflammatory/autoimmune disease RA.

## Vitamin D and Immunity

The multi-modal innate immune system is the body’s first line of defence against pathogens, comprising physical barriers (e.g. epithelium), chemical barriers (e.g. stomach acid), complement proteins and cellular responses such as those mediated by macrophage, DC and neutrophils. Vitamin D participates in several of these processes, including the maintenance of barrier function in the intestine, by regulating tight junctions [29] and intestinal epithelial cell apoptosis [30]. However, to date, studies of vitamin D and innate immunity have predominantly explored its impact on antigen-presenting cells, such as macrophages and DC, which participate in the recognition of and response to pathogen-associated molecular patterns (PAMPs) via pattern recognition receptors (PRR) [25, 26] (see Fig. 1). DNA target sequences for 1,25-(OH)_2_D3-bound to VDR, referred to as vitamin D response elements (VDRE) have been found in multiple genes associated with PRR responses, including the antibacterial proteins NOD2 [31], hepcidin antimicrobial protein (HAMP) [32], cathelicidin (CAMP) [33], B-defensin 2 (DEFB4) [33, 34] and TREM-1 [35]. Toll-Like Receptors (TLRs) are an important group of PRR, and 1,25-(OH)_2_D3 has been shown to downregulate TLR2 and TLR4 in monocytes [36], abrogating over-elaboration of TLR immune responses to PAMPs and damage-associated molecular patterns (DAMPs). In a separate study, methylation of the Vitamin D Receptor (VDR) gene and single-nucleotide polymorphisms in the VDR were also found to alter VDR-mediated TLR1/2 signalling in monocytes [37].

Vitamin D is also known to differentially regulate innate immune cell subsets, influencing cell maturation, metabolism and antigen presentation, alongside response to and production of cytokines and chemokines [13, 14, 38, 39]. Notably, mature DC express CYP27B1 but little VDR, whereas the converse is true for immature DC [13]. This has led to the hypothesis that mature DC may in fact produce vitamin D locally on activation, which then acts on immature DC to modulate immune responses [13, 40]. The principal effect of 1,25-(OH)_2_D3 on DC is to suppress maturation markers such as CD80/CD86 [41–43] and CD83 [44], increase IL-10 production and decrease pro-inflammatory cytokines [41, 45, 46]. In this way, 1,25(OH)_2_D3 promotes an immature, tolerogenic DC phenotype [47–49], thereby reducing antigen presentation to T cells.

Adaptive immune cells are also modulated directly by 1,25-(OH)_2_D3, with VDR being transiently expressed by developing thymocytes, and re-activation of VDR expression in peripheral T and B cells following immune challenge [50, 51]. 1,25-(OH)_2_D3 was initially thought to act primarily as a regulator of T and B cell proliferation, but is now known to play a more important role in regulating T cell phenotype [24, 52–54]. In particular, 1,25-(OH)_2_D3 has been shown to suppress inflammatory interleukin-17 expressing CD4+ T-helper (Th) cells (Th17) [55] and Th1 cells [56], whilst promoting differentiation of Th2 cells [57] and regulatory T cells (Treg) [58, 59] (see Fig. 1). However, vitamin D may also promote some immune responses by enhancing effector T cell responses including CD8+ cytotoxic function [53], and other studies have reported a role for 1,25-(OH)_2_D3 in promoting T cell receptor (TCR) expression and T cell activation [60]. Although less well studied, 1,25-(OH)_2_D3 has also been shown to modulate the function of other cytotoxic lymphocytes such as natural killer cells (NK), with apparent effects of NK cell activation [61]. Likewise, effects of vitamin D have been described for natural killer T cells (NKT), and NKT cell development is lost in mice lacking the *VDR* gene [62]. In contrast to the wide range of data on vitamin D and T cells, there is a scarcity of detailed studies for effects of 1,25-(OH)_2_D3 on B cells, which may include indirect effects via T cell modulation [63], and direct B cell effects on class switching [64], IL-10 production [65] and CCR10 production [66].

## Vitamin D in Autoimmune Disease

Given the proposed role for vitamin D as an immune regulator, it is perhaps unsurprising that vitamin D deficiency has been linked to both allergic [67] and autoimmune diseases [4–8, 68, 69]. The increasing incidence/prevalence of MS proportional to distance from the equator, suggests that environmental factors such as sunlight and vitamin D may be involved in disease aetiology [70]. This is further underlined by studies from the UK showing that risk of immune-related diseases is significantly influenced by the season of birth as the serum 25-OHD3 levels is associated with this [71]. Likewise, genetic variants associated with heritable effects on vitamin D status have also been linked to MS disease onset and progression [72, 73]. However, differences between genders [74], ethnic groups [75] and potentially different subtypes of MS [76], make it difficult to decipher the exact role of vitamin D in MS. Furthermore, although vitamin D supplementation has been successful in ameliorating MS symptoms in some studies [77–80], this has not been demonstrated consistently [81]. Larger randomised controlled trials (RCTs) are now needed to establish whether vitamin D supplementation can reduce risk, severity and/or progression of MS. Similar to MS, vitamin D deficiency is also prevalent in SLE [82], where low serum vitamin D has been associated with dysregulation of autophagy [83], increased circulating levels of IFNα [84], increased CD4+/CD8+ T cells and a reduction in pro-inflammatory cytokines [85]. Vitamin D intake has been shown to have variable impact on SLE, with some studies showing no effect on disease severity [86–88] whilst another study reported decreased disease activity in young adults with SLE [89]. As with MS, more research is needed to fully define optimal doses and timing of intervention for vitamin D supplementation.

T1D is another autoimmune disease with reported links to vitamin D status, with vitamin D deficiency correlating with T1D risk severity [90, 91]. A prospective, non-blinded non-randomised controlled trial has also shown that T1D patients supplemented with vitamin D and with higher serum 25-OHD3 are more likely to have improved glycemic control [92]. T1D has also been linked to single-nucleotide polymorphic variants in the *CYP27B1* [93–95] and *VDR* [96, 97] genes. In vivo treatment with the active form of vitamin D, 1,25-(OH)_2_D3, was shown to completely suppress T1D disease in the *NOD* experimental mouse [98]. In humans, clinical trials involving supplementation with vitamin D3 or the precursor molecule 1α-hydroxyvitamin D3 in T1D patients have produced mixed results; some studies have reported delay of β-cell destruction in supplemented trial groups compared to non-supplemented groups [99–101], whilst others found no significant improvements of on β-cell preservation [102, 103].

Vitamin D deficiency has also being linked to autoimmune gastrointestinal disorders such as Crohn’s disease, a major form of IBD [104–107]. The role of vitamin D as a regulator of immune function in IBD has also been studied in vivo in mouse models. Knockout of the mouse genes for VDR (*Vdr*) [108] or 1α-hydroxylase (*Cyp27b1*) [109] shows increased severity of artificially induced colitis similar to IBD. Other studies have shown that vitamin D-deficient mice have increased severity of induced colitis, with this effect being linked to aberrant innate immune responses within the gastrointestinal tract [110]. Furthermore, it has been shown that the VDR, in conjunction with 1,25-(OH)_2_D3, protects against the onset of colitis in mice by maintaining barrier function within the gastrointestinal mucosal epithelium [111]. Despite these positive studies, it is still unclear whether vitamin D deficiency is a direct cause of IBD including Crohn’s disease, or whether there is a reverse causation effect.

## Vitamin D and Rheumatoid Arthritis

Rheumatoid arthritis (RA) is a chronic inflammatory/autoimmune arthritis characterised by synovitis of peripheral joints, with extra-articular manifestations. If untreated, unopposed inflammation leads to joint destruction, loss of function and disability, and RA is also associated with premature mortality secondary, at least in part, to the effects of chronic inflammation on cardiovascular health [112]. Like other autoimmune diseases, there is growing interest in the role of vitamin D deficiency in the aetiopathogenesis of RA [9]. RA is thought to be triggered by environmental factors [113], in patients with an underlying genetic susceptibility [114, 115] leading to dysfunction of innate and adaptive immunity, tipping the balance in preference of autoimmunity over tolerance [116]. Whilst smoking is well recognised as a strong environmental risk factor other potential factors include vitamin D [9].

### Vitamin D Status and RA Disease Risk and Progress

In a recent meta-analysis and systematic review, Lin J et al. analysed 24 studies published prior to May 2015 whose focus was the relationship between serum 25-OHD3 and clinical/laboratory indices of RA disease activity. Overall, they reported an inverse relationship between serum 25-OHD3 and RA disease activity [117]. However, they also identified important differences between patient subgroups, including a stronger inverse relationship between RA disease activity and serum 25-OHD3 in studies from developing countries, and in low-latitude climates. Since the publication of this meta-analysis, further studies have been conducted. Therefore, to establish a clearer picture of whether vitamin D levels are indeed significantly lower in RA, and are linked to disease activity, all studies of vitamin D levels in RA patients currently listed on Pubmed are summarised in Table 1. The search terms “vitamin D levels” and “rheumatoid arthritis” were used, and no restrictions on study date were applied. In addition, studies included in the meta-analysis (referenced above) were also included. The collected studies listed in Table 1 underline the significant heterogeneity between studies and their findings, making it difficult to determine a clear role for vitamin D deficiency in the onset, progression and/or severity of RA. Studies to date have been conducted in over 20 countries, which invariably means that there will be confounding differences in environmental factors including sunlight exposure and diet, and genetic factors. Moreover, the overwhelming majority of the reported studies were observational or cross-sectional by design, and as such can only report on the association between RA disease and vitamin D, rather than a causal role.

**Table 1:** Summary of studies comparing serum vitamin D levels with disease activity in RA

Study details (year, lead author)	Population size and ethnicity	Disease duration	Analytical method(s)	Metabolite(s) measured	Cut off for vit D def.	Vit D lower in HC vs. RA	Association of vitamin D with disease parameter(s)^1^
1998, Oelzner	RA = 96, Germany	Mean 12.2 years (range 6 months–38 years)	RAI	1,25(OH)_2_ D_3_	Unclear	n/a	Neg: disease activity Pos; urinary collagen crosslinks ↑ DA is assoc. neg. Ca balance and ↓ bone formation
2006, Cutolo	RA = 118, HC = 75, Estonia and Italy	Not stated	RAI	25OHD	n/a	n/a	Neg: DAS-28, however, correlation varied according to time of year and country of origin
2010, Craig	RA = 266 (African Americans)	Mean 31.2 months (SD = 7.3 months)	Unclear	25OHD	< 15 ng/mL	n/a	Nil
2010, Haque	RA = 62, USA	Mean 11.6 years (SD = 12.3 years)	Standardised (Quest + Lab − corp)	25OHD	< 30 ng/mL	n/a	Neg: DAS28, pain and HAQ in active RA (DAS28 > 2.6) only
2010, Rossini	RA = 1191, HC = 1019, Italy	Mean 11.5 years (SD = 8.7 years)	ELISA	25OHD	< 30 ng/mL	No	Neg: HAQ disability, DAS28, MADLS, high Steinbrocker functional state
2011, Braun-Moscovici	Rheumatic disease = 121 (RA = 85), Israel	Mean = 9.9 years (SD = 8.5 years)	NOS	25OHD	Unclear	n/a	Nil
2011, Turhanoglu	RA = 65, HC = 40, Turkey	Mean = 7.73–7.95 years	EIA	25OHD	Not specified	No	Neg: DAS-28, CRP, HAQ
2012, Kostoglou-Athanassiou	RA = 44, HC = 44, Greece	Not stated	RAI	25OHD3	n/a	Yes	Neg: DAS-28, CRP, ESR
2012, Baker	RA = 499, USA, China	Not stated	ELISA	25OHD	< 50 nmol/L (< 30 ng/mL)	Yes	Nil
2012, Attar	RA = 100, HC = 100, Saudi Arabia	Mean 4.7 years (SD = 5 years)	LC MS/MS	25OHD	< 30 and < 10 ng/mL^	No	Neg: DAS28 ^nb study used two definitions for deficiency
2012, Baykal	RA = 55, HC = 45, Turkey	Not stated	Elecsys 25(OH)D reactive kit	25OHD	< 30 nmol/L	Yes	Nil
2012, Heidari	RA = 108, UIA = 39, HC = 239, Iran	Not stated	ELISA	25OHD	< 20 ng/mL	No	Correlation of vitamin D with RA disease parameters was not an objective of this study; the study simply compared 25OHD between disease/control
2013, Atwa	RA = 55, PsA = 43, HC = 40, Egypt	Mean 4.93 years (SD = 3.11 years)	CLA	25OHD	n/a	Yes	Nil
2013, Chen	RA = 110, HC = 110, China	Mean = 6.51yr, SD = 6.82 yr	RAI	25OHD	Not specified	n/a	Neg: DAS28
2013, Furuya	RA = 4793, Japanese	Mean = 12 years	RAI	25OHD	< 20 ng/mL	n/a	Neg: Japanese HAQ disability score, NSAID use
2013, Haga	RA = 302, Denmark	Mean = 10.5 years (range 0–50 years)	HPLC–MS	25OHD	< 50 nmol/L (< 30 ng/mL)	n/a	No assoc. overall; however, severe deficiency (< 15 nmol/L 25OHD3) was associated with increased DAS28 > 5.1, CRP, RF and ≥ 3 DMARDs
2013, Higgins	RA = 126, New Zealand	Mean = 12 years (range 1–37 years)	Immunoassay method NOS	25OHD	< 50 nmol/L (< 30 ng/mL)	n/a	Neg: VAS. This parameter of the DAS28 score alone accounted for assoc. with RA
2013, Sabbagh	Rheum dx = 56 (RA = 39), non-rheum dx = 60	Not stated	NOS	25OHD	< 50 nmol/L (< 30 ng/mL)	Yes	Neg: DAS28-ESR
2013, Yazmalar	RA = 71, AS = 72, OA = 74, HC = 70, Turkey	Not stated	HPLC	25OHD	n/a	No	Nil
2014, Cote	RA = 120, HC = 1341, USA	Not stated	RAI or LC MS/MS	Vit D	< 20 ng/mL + < 30 ng/mL	n/a	Nil assoc. between vit D and RA onset
2014, Gheita	RA = 63, HC = 62, Egypt	Mean = 5.89 years (SD = 3.67)	CLA	25OHD	< 20 ng/mL	Yes	Neg: QoL, HAQ II, FMS *RA *+* FMS had lower vit D than RA alone*
2014, Hong	RA = 130, HC = 80	Mean 6 years (range 2 months–40 years)	ELISA	25OHD	n/a	Yes	Neg: SJC, TJC, joint pain, EMS, HAQ, Plt, ESR, IL-17, IL-23
2014, Hiraki	Pre-RA = 166, HC = 490	n/a	RAI	25OHD	n/a	n/a	Nil association found between 25OHD and development of RA, except in a small subset of females just prior to RA onset
2014, Sahebari	RA = 99, HC = 68, Iran	Mean = 59 years (SD 5.6 years; range 0.2–20 years)	ELISA	25OHD	< 30 nmol/L	No	Nil; however, all patient, were on vit D replacement
2014, Sharma	RA = 80, HC = 80	Not stated	ELISA	25OHD	< 10 ng/mL	Yes	Neg: DAS28
2015, Cooles	RA = 73, UA = 40, OA = 58, NIA = 89, other IA = 50, ReA = 14, CrA = 19	RA—49 years (range 18–88)	Not stated	25OHD	n/a	No	Nil
2015, Raczkiewicz	RA = 97, OA = 28, Poland	5.8 ± 5.4 years (vit D > 20 ng/dL) 8.8 ± 9.8 years (vit D < 20 ng/dL)	CLA	25OHD	< 20 ng/dL	n/a	Neg: DAS28, HAQ, BDI Pos: PA, SF-36* * remained sig. after multivariate analysis
2015, Matsumoto	RA = 181, HC = 186, Japan	Mean = 10.2 years (5.2–20 years)	RAI	25OHD	Not specified	Yes	Nil
2015, Azzeh	RA = 102, Saudi	Not stated	CLA	25OHD	< 30 ng/mL	n/a	Neg: DAS28
2015, Brance	RA = 34, HC = 41, Argentina	Mean = 7.6 years (SD = 1.4 years)	CLA	25OHD	< 20 ng/mL (< 50 nmol/L)	Yes	Neg: DAS-28
2015, Cen	RA = 116, China	Not stated	ELISA	25OHD	< 50 nmol/L (< 30 ng/mL)	Yes	Nil
2015, Wang	Early RA = 154, HC = 60, China	Disease duration < 1 year	CLA	25OHD	<20 ng/mL	Yes	Neg: ACPA, ESR, DAS
2016, Cecchetti	RA = 894, HC = 861, multiple countries	Not available	NOS	25OHD	≤ 10 ng/mL	Yes	Neg: DAS28-CRP, SDAI, CDAI
2016, Pakchotanon	RA = 239, Thai	Median 84 months (range 48–132 months)	CLA	25OHD_2_ 25OHD_3_	n/a	n/a	Nil
2016, Zakeri	RA = 66, Iran	Not stated	CLA	25OHD	n/a	n/a	Neg: DAS-ESR, SJC, TJC, GHS, EMS
2017, Mateen	RA = 100, HC = 50	Not stated	CLA	25OHD	n/a	Yes	Neg: TNF-α, IL-1β, IL-6, IL-10, IL-17, ROS
2017, Hajjaj-Hassouni	RA = 1413, 15 countries	8.3 years (range 3.6–15.2 years)	NOS	25OHD	≤ 10 ng/mL	n/a	Neg: DAS + Corticosteroid dose
2017, Vojinovic	RA = 625, HC = 276,13 European countries	Mean = 11 years (SD = 9 years)	CLA	25OHD	< 20 ng/mL	Yes	Neg: DAS28-CRP, RAID, HAQ, SRS/HRS/GRS domains of D-PRO
2018, Herly	RA = 160, Denmark	Median 14.1 weeks (range 6.1–26.6)	LC MS/MS ………. RAI	25OHD_2_ 25OHD_3_ ………. 1,25(OH)_2_ D	< 50 nmol/L ……….	n/a ……….	Nil Nil ……………………….. Neg: DAS28-CRP, HAQ, CRP, VAS-pain Pos: ACPA
2018, de la Torre Lossa	RA = 100, Ecuador	Full article in Spanish	Full article in Spanish	25OHD	Full article in Spanish	Full article in Spanish	Nil
2018, Khoja	RA = 41, HC = 41	Not available	Unclear	Unclear	Unclear	Yes	Neg: PROs

References are shown with Supplementary Table 1

*ACPA* anti-citrullinated peptide antibody, *CDAI* clinical disease activity index, *CLA* chemiluminescent assay, *DA* disease activity, *DAS28* disease activity score 28, *ELISA* enzyme-linked immunosorbent assay, *EMS* early morning stiffness, *FMS* fibromyalgia syndrome, *GHS* global health score, *GRS* global risk score (SRS + HRS), *HAQ* health assessment questionnaire, *HRS* habitus risk score, *LC MS/MS* liquid chromatography tandem mass spectrometry, *Neg* negative correlation between vitamin D and outcome measure, *NOS* Not otherwise specified, *OA* osteoarthritis, *Pos* positive correlation between vitamin D and outcome measure, *PROs* patient-reported outcomes, *PsA* psoriatic arthritis, *RA* rheumatoid arthritis, *HC* healthy control, *RAI* Radioimmunoassay, *ROS* reactive oxygen species, *SDAI* simple disease activity index, *SJC* swollen joint count, *SRS* symptom risk score, *TCJ* tender joint count, *VAS* visual analogue score

RA is a heterogeneous disease, yet few studies have considered analysing RA subgroups based on antibody status, disease severity or duration. Where subgroups have been defined, this is often based on disease activity score 28 (DAS-28); a composite score that captures different subjective and objective measures of disease activity, hence the potential for skewing of results by one component. One study that did address this reported no correlation between vitamin D levels and DAS-28, with or without the inclusion of the patient global visual analogue scale score (Patient global VAS)—a scale for evaluating the patient’s overall perception of their RA activity. However, patient global VAS on its own did correlate with serum vitamin D levels [118]. In 2015, Cooles et al. reported data showing the relationship between serum 25-OHD3 levels and various clinical parameters in early RA (median symptom duration 12 weeks, range 4–104 weeks) [119]. They found no clear association between 25-OHD3 and early RA, but in early osteoarthritis (OA) 25-OHD3 was inversely associated with global health visual analogue scale scores (GH-VAS). This suggests that OA, which commonly co-exists with RA, may act as a confounder in interpreting the relationship between vitamin D and RA. These observations are intriguing, and need to be further explored to determine the role, if any, for vitamin D in pain and fatigue associated with RA.

Relatively few studies have assessed the impact of RA treatment regimens on the apparent inverse correlation between serum 25-OHD3 and RA disease activity [120–122]. Treatment for RA is aimed at reducing disease activity, and therefore comparing serum 25-OHD3 levels with measures of disease activity after the initiation of treatment could mask prior effects of vitamin D deficiency on treatment naïve disease. Since the 1990s, an early and aggressive approach to the management of new RA has been widely used in order to maximise chances of inducing remission [123]. Today, this strategy is fully integrated in clinical practice, but its application in the context of low vitamin D, and vitamin D replacement, for treatment naive, newly diagnosed RA patients, is still unclear.

As well as clinical and biochemical differences in subgroups of RA, there are also likely genetic differences which may influence the relative importance of vitamin D in the immunopathogenesis of disease. For example, Dennis et al. identified four distinct synovial tissue gene expression profiles in a cohort of RA patients [124]. Although some of these differences in gene expression may be related to the patient’s disease duration or treatment regimen, it seems unlikely that this sufficiently explains all the observed differences, and that distinctly different gene signatures may indeed characterise different subsets of RA. Accordingly, it seems plausible that vitamin D deficiency may play slightly different roles in the aetiopathogenesis and progression of disease in different groups of patients. Ultimately, the role of vitamin D in RA appears far too complex to be understood through simple observational methods.

To date, studies of RA disease and vitamin D have focused on the link between RA and serum levels of the main circulating form of vitamin D, 25-OHD3. However, 25OHD3 is an inactive precursor for 1,25-(OH)_2_D3 and therefore has limited functional relevance for immunomodulation. Consequently, there has been a revival of interest in the role of other vitamin D metabolites both in serum and disease-affected synovial fluid as potentially more informative markers for vitamin D function in RA. Research on this subject began more than 25 years ago with seminal studies of vitamin D metabolism in RA tissues [125–127]. These reports indicated that concentrations of 25-OHD3 were significantly lower in synovial fluid relative to paired patient blood, but also revealed significant capacity for the generation of 1,25-(OH)_2_D3 by macrophages isolated from synovial fluid [127]. In more recent studies, we have measured multiple vitamin D metabolites—the vitamin D ‘metabolome’ in paired serum and synovial fluid from patients with established RA, reactive arthritis and healthy controls using the current gold standard for vitamin D analysis, liquid chromatography–tandem mass spectrometry (LC–MS/MS) [128]. These studies showed that for markers such as swollen joint count (SJC), synovial fluid levels of vitamin D metabolites correlated better with RA disease activity than their circulating serum counterparts [128].

Another area of contention for vitamin D and RA concerns the definition of vitamin D deficiency itself (Table 1). In the UK, the National Institute of Clinical Excellence (NICE) recommends diagnosis of vitamin D deficiency when serum 25-OHD3 is < 30 ng/mL, and states that for some people 30-50 ng/mL may be insufficient, citing recommendations from the national osteoporosis society guidelines [129, 130]. Hence, there appears to be an ambiguity around whether or not some patients need more vitamin D than others. Current NICE guidance does not define what constitutes adequate vitamin D status in patients of different ages, sex, ethnicities or disease states; for example, patients at risk of RA vs patients with established RA, or in other inflammatory diseases. To date, few studies have attempted to define what adequate supplementation means in RA. A cohort study published in 2012 observed that even supplementation 800-880 IU/day did not achieve adequate repletion of vitamin D (defined as > 20 ng/mL) in 27.7% of RA patients who were vitamin D-deficient, although duration of therapy was not reported [131]. This suggests that different levels of vitamin D replacement may be required in different RA patients, depending on pre-supplementation of vitamin D levels, sunlight exposure and skin colour (darker skin absorbs less UV light to make vitamin D). Failure to adequately replete RA patients could also be related to poor treatment compliance or inadequate duration of supplementation. Importantly, inadequate repletion was related to higher HAQ scores for RA patients, implying that inadequate improvement of vitamin D status following supplementation had poorer outcomes [131]. Alternatively higher disease activity may simply be associated with less time spent outdoors, indirectly impacting sunlight exposure and skin synthesis of vitamin D. Further RCTs are needed to more clearly define the optimal levels of vitamin D for patients with RA, and how to achieve and maintain these levels. The next section of the review describes the reported supplementation trials for vitamin D and RA.

### Vitamin D Supplementation Trials in RA

Ultimately, defining vitamin D deficiency in the context of RA is of clinical interest only if replacing vitamin D in RA patients who are deficient is likely to improve disease symptoms, or even prevent the onset of RA in those at risk. To date, vitamin D supplementation trials for RA have varied appreciably in terms of patient numbers, characteristics, disease duration and severity, concomitant medication regimen, type of/duration of supplementation regimen, number of outcomes and period over which these outcomes were measured (Table 2). A recent meta-analysis identified 9 RCTs of vitamin D supplementation for ≥ 3 months in rheumatic diseases, including 5 studies of RA patients [132]. In RA, there was a decrease in the rate of disease flare, VAS and DAS-28 with vitamin D supplementation; however, all failed to reach statistical significance. Similar findings have also been reported in previous meta-analyses on this subject [133]. Conversely, a meta-analysis conducted in 2012 found that all but one of the 11 studies included in the meta-analysis suggested low vitamin D intake was linked with both increased risk of RA and greater disease activity [134]; however, the studies included in that analysis were cohort/association by design, and not RCTs. Challenges associated with identifying whether vitamin D supplementation has a beneficial effect in RCTs to date include inter-study heterogeneity and relatively small sample numbers for a meta-analysis, with only 5 RCTs included, thus emphasising the need for larger RCTs in different subsets of RA patients to fully elucidate the role, if any, for vitamin D supplementation in the management of RA. In addition, there may be differences in vitamin D-binding protein levels, and other genetic variants, which influence the efficacy of vitamin D supplementation [135]. Vitamin D supplementation in low/moderate doses is not thought to be harmful to patients, has wider health benefits, is relatively inexpensive and has fewer side effects/interactions compared with many other commonly used treatments for RA, such as non-steroidal anti-inflammatory drugs (NSAIDs), or conventional synthetic or biological disease-modifying anti-rheumatic drugs (DMARDs). Evidence is also emerging that vitamin D may augment certain therapies in RA. In one in vitro study, vitamin D 1,25-(OH)_2_D3 was shown to act synergistically with the biologic drug abatacept to inhibit T cell activation driven by anti-CD3 cross-linking, and promote a pro-regulatory CD28 phenotype [136]. The potential for enhancing the effects of biologics with simple, low-risk addition of 1,25-(OH)_2_D3 is interesting, and further work is required to validate this initial in vitro finding.

**Table 2:** Vitamin D supplementation trials in rheumatoid arthritis

Study	Study participants (no. eligible + DMARD tx)	Treatment groups/trial design/BL vit D	Primary and secondary outcome measures	Summary of key findings
Andjelkovic et al. (1999)	RA = 19 (on MTX ± GC, active dx)	2 microg/day oral alfacalcidol for 3/12 in 2 groups; mod + highly active RA. Control group = same patients data collected over 3 months prior to suppl Open-label trial	ESR, CRP, EMS, Richie index, Lee index at 3 months	CRP, SJC, TJC, Richie index and Lee index all significantly decreased after 3/12 RF and CRP were decreased, but this was not statistically significant
Gopinath et al. (2011)	RA = 121 (on triple DMARDs)	500 IU 1,25OH2D3 + CaCO3 vs. CaCO3 Open-label 25OHD3 < 20 ng/mL at BL	Pain relief assessed by patient VAS at first relief of pain and again at 3/12	No difference in time achieves first pain relief; however, there was higher pain relief in the vit D group at 3/12 (NNT = 5)
Salesi et al. (2012)	RA = 117 (on MTX ± HCQ/CQ, active dx)	50,000 IU/week for 3 months vs. placebo Double-blinded trial	>0.6 or > 1.2 improvement in DAS28 at wk 12	No improvement in outcome measures reported
Dehghan et al. 2014	RA = 80 (remission for 2/12)	Cholecalciferol 50,000 IU/week versus placebo Double-blind RCT 25OHD levels were < 30 ng/mL at BL	DAS28 as a marker of relapse, over 6/12	No statistical significant reduction in relapse rate was observed
Yang J et al. (2015)	RA = 377 (RA in remission)	Alfacalcidol 0.25 microg BD for 24 months in vit D def. RA vs. placebo vs. RA with normal vit D levels and no treatment Open-label Deficiency = 25OHD3 < 30 ng/mL	VAS, SHC, TJC, CRP, ESR and DAS-28 every 2-3/12	Normal vit D assoc. with ↓recurrence. No difference was observed with or without vit D suppl. In RA with low vit D
Buondonno et al. (2017)	Early RA = 39 (Tx naïve), HC = 31	MTX + GC vs. MTX + GC + 300,000 IU (one-off dose) Double-blind RCT	T cell phenotypes, OC precursors, inflammatory cytokines, clinical parameters at 3/12	Reduced IL-23, incr. GHS reported in the vit D suppl. group
Chandrashekara et al. (2017)	RA = 73 (on DMARDs, active dx)	60, 000 IU/week for 6 weeks then 60,000 IU/month for 3/12 Open-label 25OHD3 < 20 ng/mL at BL + DAS28-CRP > 2.6	Improvement in DAS28-CRP, vitamin D status	↓ DAS28-CRP and ↑ vit D > 20 ng/mL in the tx group

References are shown with Supplementary Table 2

*BD* twice daily, *BL* baseline, *CQ* chloroquine, *CRP* C-reactive protein, *DAS* disease activity score, *DMARDs* disease-modifying anti-rheumatic drugs, *EMS* early morning stiffness, *ESR* erythrocyte sedimentation rate, *GC* glucocorticoids, *HCQ* hydroxychloroquine, *MTX* methotrexate, *NNT* number needed to treat, *RA* rheumatoid arthritis, *RF* rheumatoid factor, *SJC* swollen joint count, *TJC* tender joint count, *Tx* treatment, *VAS* visual analogue scale

### Cellular Targets for Vitamin D in RA

The pathogenesis of RA involves both innate and adaptive immune activities. Adaptive CD4+ T cells are critical in the pathogenesis of RA. For example, T cells are a source of RANKL, leading to osteoclast activation and subsequent joint destruction in RA [137]. However, antigen-presenting cells (APCs) such as DC also contribute to RA by providing the necessary co-stimulatory signals required for CD4+ T cell activation [138, 139]. Not only do APCs activate T cell proliferation, but they also influence T cell phenotype (Th1/Th2/Th17/Treg) and subsequent cytokine profile, and Th1/17 are both known to be relevant to the pathogenesis of RA [140, 141]. With this in mind, it is clear that the immunomodulatory activities of 1,25-(OH)_2_D3 described earlier in the review (see Fig. 1) have the potential to influence both the innate and adaptive immune cell types that contribute to the dysregulated immunity associated with RA. In a murine model of RA, tolerogenic DCs (tolDCs) were observed to reduce severity and progression of RA disease by increasing IL-10-producing T cell numbers and reducing Th17 cell counts [142]. It is therefore interesting to note that 1,25-(OH)_2_D3 can induce tolDC [143], and tolDCs, generated ex vivo using 1,25-(OH)_2_D3 have been proposed as a potential strategy for RA therapy [144]. In this instance, 1,25-(OH)_2_D3 was used in combination with the glucocorticoid dexamethasone which is known to promote a tolDC phenotype [58].

In addition to enhanced activity of Th1 and Th17 cells, RA disease is also characterised by reduced Treg activity, including decreased Treg numbers [145], a reduction in Treg: Th1/Th17 ratio [146], altered Treg function [147] and differences in Treg number and function in the peripheral blood compared with the synovium [148]. However, in some studies, numbers of circulating Treg in RA were similar to those found in healthy controls [149] and osteoarthritis patients [150]. Tregs are likely to be a key target for vitamin D in RA. In animal models of experimental autoimmune encephalitis (the most widely used mouse model of MS), IBD and T1D, 1,25-(OH)_2_D3 promotes a Treg phenotype and augment IL-10 production, thus inhibiting Th17 responses and ameliorating disease [54]. In studies using human Tregs, the 1,25-(OH)_2_D3 analogue TX527 skewed the Treg cell phenotype in favour of migration to sites of inflammation [151], whilst also promoting a stable Treg phenotype [152]. The growing pool of ex vivo and in vitro evidence linking vitamin D and Treg function now requires replication in vivo, particularly in diseases such as RA.

To date, most studies of the T cell actions of 1,25-(OH)_2_D3 have been based on the analysis of mixed populations of circulating T cells from healthy donors. However, in recent studies, we have shown that T cells from synovial fluid of RA patients’ inflamed joints are relatively insensitive to 1,25-(OH)_2_D3, despite expressing the VDR machinery required for 1,25-(OH)_2_D3 signalling [153]. This is due, in part, to decreased 1,25-(OH)_2_D3 responsiveness in the memory T cells that predominate in RA synovial fluid, but also involves tissue-specific effects, with synovial fluid memory T cells showing decreased responses to 1,25-(OH)_2_D3 relative to peripheral blood memory T cells [153]. Based on these observations, we have proposed that immunomodulatory responses to vitamin D at tissue sites of inflammation are impaired by target cell insensitivity to 1,25-(OH)_2_D3. If this is the case, then conventional analysis of the effects of vitamin D using circulating blood immune cells can only provide a limited picture of immunomodulation by vitamin D in diseases such as RA. Likewise, to overcome the vitamin D-insensitivity observed in RA patient, inflamed joints may require alternative strategies. This could include the use of higher doses of vitamin D supplements to enhance localised tissue levels of 1,25-(OH)_2_D3, or the use of vitamin D as an adjunct to other RA therapies. With respect to the latter, we have recently shown that 1,25-(OH)_2_D3 can more potently inhibit T cell activation when used in combination with the CD28 co-stimulatory blocker abatacept [154].

Beyond the actions of innate and adaptive immune cells, synovial fibroblasts (synoviocytes) also play an important role in the pathogenesis of RA. In studies using the immortalised synoviocyte cell line, MH7A, 1,25-(OH)_2_D3 has been shown to promote synoviocyte apoptosis, which might protect against RA, but only when cells were treated with both tumor necrosis factor α (TNFα) and 1,25-(OH)_2_D3 [155]. These observations suggest that TNFα is required for 1,25-(OH)_2_D3 to have anti-inflammatory effects in RA, which is a paradoxical observation given the use of TNFα inhibitors as biological treatment in RA. Conversely, other studies have reported synergistic effects of TNFα inhibition and 1,25-(OH)_2_D3 in suppressing Th17-mediated inflammation [156], suggesting a complex interaction between TNFα and 1,25-(OH)_2_D3. Other studies using MH7A cells have shown synergistic effects of interleukin 1β (IL-1β) and 1,25-(OH)_2_D3 in suppressing the production of IL-6 and TNFβ levels, and Th17-inducing cytokines (IL-1β, IL-6 and IL-23) synergistically enhanced the pro-regulatory effect of 1,25-(OH)_2_D3 on T cell phenotype [136], further emphasising important interactions between 1,25-(OH)_2_D3 and pro-inflammatory cytokines in RA [157]. Collectively, these observations suggest that the beneficial effects of 1,25-(OH)_2_D3 are most potent when the threshold for activation of the immune response is breached, leading to concomitant production of inflammatory cytokines. Therefore, in the setting of inflammation, vitamin D appears to function as a negative-feedback regulator, attenuating the inflammatory immune responses. Vitamin D may also influence the synovial microenvironment by modulating factors that influence joint bone and cartilage damage. In studies using synoviocytes and articular chondrocytes from RA patients, 1,25-(OH)_2_D3 was shown to regulate matric metalloproteinases and prostaglandins, but only in the presence of IL-1β [158], suggesting, as outlined earlier, that 1,25-(OH)_2_D3 is only effective as a regulator of synoviocytes in the setting of RA disease inflammation.

## Conclusions

Future studies of vitamin D and RA are needed firstly to expand our current understanding of the mechanisms by which 1,25-(OH)_2_D3 is able to regulate key cells associated with RA. In particular, the observation that T cells from the inflamed joints of RA patients are insensitive to 1,25-(OH)_2_D3 [153] indicates that RA disease is associated with a corruption of vitamin D signalling that may be fundamentally important for RA disease pathology, and the therapeutic use of vitamin D. A key question that remains to be answered is whether vitamin D has greater benefits in protecting against the onset of RA as opposed to its potential application as a therapy for established RA disease. Thus, future studies to assess the effects of vitamin D supplementation on disease prevention in individuals at risk of RA, and disease development in those at early stages of RA, are required. These studies are likely to be informed by recent meta-analyses for immune effects of vitamin D. Notably, the observation from the analysis of acute respiratory infection trials that vitamin D supplementation was more beneficial in patients with low baseline serum vitamin D, and was more effective when supplementation was used as lower daily or weekly dosing [159], provides some important pointers for future studies of vitamin D and RA. Repeated lower doses of vitamin D supplementation would also help to avoid potential adverse effects of higher doses of vitamin D, in particular the reported increased risk of falls in elderly patients receiving a single bolus of higher-dose vitamin D [160]. Future studies also need to take into consideration clinical subgroups of patients, including distinguishing between ethnic groups and disease of different durations. The potential for a simple, low-risk and low-cost intervention such as vitamin D as a plausible adjunctive treatment for RA is an exciting notion. Robust evidence to support wider routine use of vitamin D supplementation in RA has the potential to significantly enhance treatment for RA and other autoimmune diseases.

## Electronic supplementary material

Below is the link to the electronic supplementary material.

Supplementary material 1 (DOC 123 kb)
