# Supplementary material for: Vitamin D, Autoimmune Disease and Rheumatoid Arthritis
Source: Calcif Tissue Int. 2019 Jul 8;106(1):58–75. doi: 10.1007/s00223-019-00577-2 (PMC6960236; doi:10.1007/s00223-019-00577-2)
Supplement: Supplementary file 1 — Supplementary material 1 (DOC 123 kb) [file 223_2019_577_MOESM1_ESM.doc]

**Supplementary Table 1.** Summary of studies comparing vitamin D levels with disease activity in RA

| **Study details (year, lead author)** | **Population size and ethnicity** | **Disease Duration** | **Analytical Method(s)** | **Metabolite(s) measured** | **Cut off for vitD def.** | **Vit D lower in HC vs. RA** | **Association of vitamin D with disease parameter(s)¹** |
| --- | --- | --- | --- | --- | --- | --- | --- |
| 1998, Oelzner | RA=96, Germany | Mean 12.2 yrs (range 6m – 38yrs) | RAI | 1,25(OH)_2_ D_3_ | Unclear | n/a | Neg: disease activity  Pos; urinary collagen crosslinks  ↑ DA is assoc. neg. Ca balance and ↓ bone formation |
| 2006, Cutolo | RA=118, HC=75, Estonia and Italy | Not stated | RAI | 25OHD | n/a | n/a | Neg: DAS-28, however correlation varied according to time of year and country of origin |
| 2010, Craig | RA=266 (African Americans) | Mean 31.2m (SD= 7.3m) | Unclear | 25OHD | <15ng/ml | n/a | nil |
| 2010, Haque | RA=62, USA | Mean 11.6yrs (SD= 12.3yrs) | Standardised (Quest + Lab– corp) | 25OHD | <30ng/ml | n/a | Neg: DAS28, pain and HAQ in active RA (DAS28 >2.6) only |
| 2010, Rossini | RA=1191, HC=1019, Italy | Mean 11.5yrs (SD= 8.7yrs) | ELISA | 25OHD | <30ng/ml | No | Neg: HAQ disability, DAS28, MADLS, high Steinbrocker functional state |
| 2011, Braun-Moscovici | Rheumatic disease=121 (RA=85), Israel | Mean = 9.9 yrs (SD= 8.5yrs) | NOS | 25OHD | Unclear | n/a | nil |
| 2011, Turhanoglu | RA=65, HC=40, Turkey | Mean = 7.73-7.95yrs | EIA | 25OHD | Not specified | No | Neg: DAS-28, CRP, HAQ |
| 2012, Kostoglou-Athanassiou | RA=44, HC=44, Greece | Not stated | RAI | 25OHD3 | n/a | Yes | Neg: DAS-28, CRP, ESR |
| 2012, Baker | RA=499, USA, China | Not stated | ELISA | 25OHD | <50nmol/L  (<30ng/ml) | Yes | nil |
| 2012, Attar | RA=100, HC=100, Saudi Arabia | Mean 4.7yrs (SD=5yrs) | LC MS/MS | 25OHD | <30 and <10 ng/ml^ | No | Neg: DAS28  ^nb study used two definitions for deficiency |
| 2012, Baykal | RA=55, HC=45, Turkey | Not stated | Elecsys 25(OH)D reactive kit | 25OHD | <30nmol/L | Yes | nil |
| 2012, Heidari | RA=108, UIA=39, HC=239, Iran | Not stated | ELISA | 25OHD | <20ng/ml | No | Correlation of vitamin D with RA disease parameters was not an objective of this study; the study simply compared 25OHD between disease/ control |
| 2013, Atwa | RA=55, PsA=43, HC=40, Egypt | Mean 4.93yrs (SD = 3.11yrs) | CLA | 25OHD | n/a | Yes | nil |
| 2013, Chen | RA=110, HC=110, China | Mean = 6.51yr, SD= 6.82yr | RAI | 25OHD | Not specified | n/a | Neg: DAS28 |
| 2013, Furuya | RA=4793, Japanese | Mean = 12 yrs | RAI | 25OHD | <20ng/ml | n/a | Neg: Japanese HAQ disability score, NSAID use |
| 2013, Haga | RA=302, Denmark | Mean = 10.5yrs (range= 0-50yrs) | HPLC-MS | 25OHD | <50nmol/L  (<30ng/ml) | n/a | No assoc. overall however severe deficiency (<15nmol/l 25OHD3) was associated with increased DAS28 >5.1, CRP, RF and ≥3 DMARDs |
| 2013, Higgins | RA=126, New Zealand | Mean = 12yrs (range 1-37yrs) | Immunoassay method NOS | 25OHD | <50nmol/L  (<30ng/ml) | n/a | Neg: VAS. This parameter of the DAS28 score alone accounted for assoc. with RA |
| 2013, Sabbagh | Rheum dx=56 (RA=39), non-rheum dx = 60 | Not stated | NOS | 25OHD | <50nmol/L (<30ng/ml) | Yes | Neg: DAS28-ESR |
| 2013, Yazmalar | RA=71, AS=72, OA=74, HC=70, Turkey | Not stated | HPLC | 25OHD | n/a | No | nil |
| 2014, Cote | RA=120, HC=1341, USA | Not stated | RAI or LC MS/MS | Vit D | <20ng/ml + <30ng/ml | n/a | Nil assoc. between vit D and RA onset |
| 2014, Gheita | RA=63, HC=62, Egypt | Mean = 5.89yrs (SD=3.67) | CLA | 25OHD | <20ng/mL | Yes | Neg: QoL, HAQ II, FMS  *RA + FMS had lower vit D than RA alone* |
| 2014, Hong | RA=130, HC=80 | Mean 6yrs (range 2m– 40yrs) | ELISA | 25OHD | n/a | Yes | Neg: SJC, TJC, joint pain, EMS, HAQ, Plt, ESR, IL-17, IL-23 |
| 2014, Hiraki | Pre-RA=166, HC=490 | n/a | RAI | 25OHD | n/a | n/a | Nil association found between 25OHD and development of RA, except in a small subset of females just prior to RA onset. |
| 2014, Sahebari | RA=99, HC=68, Iran | Mean = 59yrs (SD 5.6yrs; range 0.2-20yrs) | ELISA | 25OHD | <30nmol/L | No | nil- however all patient were on vitD replacement |
| 2014, Sharma | RA=80, HC=80 | Not stated | ELISA | 25OHD | <10 ng/ml | Yes | Neg: DAS28 |
| 2015, Cooles | RA=73, UA=40, OA=58, NIA=89, other IA=50, ReA=14, CrA=19 | RA – 49years (range 18-88) | Not stated | 25OHD | n/a | No | nil |
| 2015, Raczkiewicz | RA=97, OA=28, Poland | 5.8 ± 5.4yrs (vit D >20ng/dL)  8.8 ± 9.8yrs (vitD <20ng/dL) | CLA | 25OHD | <20ng/dL | n/a | Neg: DAS28, HAQ, BDI  Pos: PA, SF-36*  ** remained sig. after multivariate analysis* |
| 2015, Matsumoto | RA=181, HC=186, Japan | Mean = 10.2yrs (5.2-20yrs) | RAI | 25OHD | Not specified | Yes | nil |
| 2015, Azzeh | RA=102, Saudi | Not stated | CLA | 25OHD | <30ng/ml | n/a | Neg: DAS28 |
| 2015, Brance | RA=34, HC=41, Argentina | Mean= 7.6yrs (SD= 1.4yrs) | CLA | 25OHD | <20ng/ml (<50nmol/l) | Yes | Neg: DAS-28 |
| 2015, Cen | RA=116, China | Not stated | ELISA | 25OHD | <50nmol/L  (<30ng/ml) | Yes | nil |
| 2015, Wang | Early RA=154, HC=60, China | Disease duration <1yr | CLA | 25OHD | <20ng/ml | Yes | Neg: ACPA, ESR, DAS |
| 2016, Cecchetti | RA=894, HC=861, multiple countries | Not available | NOS | 25OHD | ≤10 ng/mL | Yes | Neg: DAS28-CRP, SDAI, CDAI |
| 2016, Pakchotanon | RA=239, Thai | Median = 84m (range= 48-132m) | CLA | 25OHD_2_  25OHD_3_ | n/a | n/a | nil |
| 2016, Zakeri | RA=66, Iran | Not stated | CLA | 25OHD | n/a | n/a | Neg: DAS-ESR, SJC, TJC, GHS, EMS |
| 2017, Mateen | RA=100, HC=50 | Not stated | CLA | 25OHD | n/a | Yes | Neg : TNF-α, IL-1β, IL-6, IL-10, IL-17, ROS |
| 2017, Hajjaj-Hassouni | RA=1413, 15 countries | 8.3yrs (range 3.6-15.2yrs) | NOS | 25OHD | ≤10 ng/mL | n/a | Neg: DAS + Corticosteroid dose |
| 2017, Vojinovic | RA=625, HC=276,  13 European countries | Mean=11yrs (SD=9 yrs) | CLA | 25OHD | <20ng/ml | Yes | Neg: DAS28-CRP, RAID, HAQ, SRS/HRS/GRS domains of D-PRO |
| 2018, Herly | RA=160, Denmark | Median= 14.1 weeks (range= 6.1-26.6) | LC MS/MS  ……….  RAI | 25OHD_2_  25OHD_3_  ……….  1,25(OH)_2_ D | <50nmol/L  ………. | n/a  ………. | nil  nil  ………………………..  Neg: DAS28-CRP, HAQ, CRP, VAS-pain  Pos: ACPA |
| 2018, de la Torre Lossa | RA=100, Eucador | Full article in Spanish | Full article in Spanish | 25OHD | Full article in Spanish | Full article in Spanish | nil |
| 2018, Khoja | RA=41, HC=41 | Not available | Unclear | Unclear | Unclear | Yes | Neg: PROs |

ACPA: anti-citrullinated peptide antibody; CDAI: clinical disease activity index; CLA: chemiluminescent assay; CrA: crystal arthropathy; DA: disease activity; DAS28: disease activity score 28; ELISA: Enzyme-linked immunosorbent assay; EMS: early morning stiffness; FMS: fibromyalgia syndrome; GHS: global health score; GRS: global risk score (SRS + HRS); HAQ: health assessment questionnaire; HC: healthy control; HRS: habitus risk score; IA: inflammatory arthritis; LC MS/MS: liquid chromatography tandem mass spectrometry; NIA: non-inflammatory arthralgia (fibromyalgia, etc.); Neg: negative correlation between vitamin D and outcome measure; NOS: Not otherwise specified; OA: osteoarthritis; Pos: positive correlation between vitamin D and outcome measure; PROs: patient reported outcomes; PsA: psoriatic arthritis; RA: rheumatoid arthritis; RAI: Radioimmunoassay; ROS: reactive oxygen species; SDAI: simple disease activity index; SJC: swollen joint count; SRS: symptom risk score; TCJ: tender joint count; VAS: visual analogus score

**References**

1. Oelzner P, Müller A, Deschner F, Hüller M, Abendroth K, Hein G, et al. Relationship between disease activity and serum levels of vitamin D metabolites and PTH in rheumatoid arthritis. Calcif Tissue Int. 1998;62(3):193–8.
2. Cutolo M, Otsa K, Laas K, Yprus M, Lehtme R, Secchi ME, et al. Circannual vitamin D serum levels and disease activity in rheumatoid arthritis: Northern versus Southern Europe. Clin Exp Rheumatol. 2006;24:702–4.
3. Craig SM, Yu F, Curtis JR, Alarcon GS, Conn DL, Jonas B, et al. Vitamin D Status and Its Associations with Disease Activity and Severity in African Americans with Recent-onset Rheumatoid Arthritis. J Rheumatol. 2010;37:275–81.
4. Haque UJ, Bartlett SJ. Relationships among vitamin D, disease activity, pain and disability in rheumatoid arthritis. Clin Exp Rheumatol 2010;28:745–7.
5. Rossini M, Maddali Bongi S, La Montagna G, Minisola G, Malavolta N, Bernini L, et al. Vitamin D deficiency in rheumatoid arthritis: prevalence, determinants and associations with disease activity and disability. Arthritis Res Ther. 2010;12:R216.
6. Braun-Moscovici Y, Toledano K, Markovits D, Rozin A, Nahir AM, Balbir-Gurman A. Vitamin D level: is it related to disease activity in inflammatory joint disease? Rheumatol Int. 2011;31:493–9.
7. Turhanoğlu AD, Güler H, Yönden Z, Aslan F, Mansuroglu A, Ozer C. The relationship between vitamin D and disease activity and functional health status in rheumatoid arthritis. Rheumatol Int. 2011;31:911–4.
8. Kostoglou-Athanassiou I, Athanassiou P, Lyraki A, Raftakis I, Antoniadis C. Vitamin D and rheumatoid arthritis. Ther Adv Endocrinol Metab. 2012;3:181–7.
9. Baker JF, Baker DG, Toedter G, Shults J, Von Feldt JM, Leonard MB. Associations between vitamin D, disease activity, and clinical response to therapy in rheumatoid arthritis. Clin Exp Rheumatol. 2012;30:658–64.
10. Attar SM. Vitamin D deficiency in rheumatoid arthritis. Prevalence and association with disease activity in Western Saudi Arabia. Saudi Med J. 2012;33:520–5.
11. Baykal T, Senel K, Alp F, Erdal A, Ugur M. Is there an association between serum 25-hydroxyvitamin D concentrations and disease activity in rheumatoid arthritis? Bratisl Lek Listy. 2012;113:610–1.
12. Heidari B, Hajian-Tilaki K, Heidari P. The status of serum vitamin D in patients with rheumatoid arthritis and undifferentiated inflammatory arthritis compared with controls. Rheumatol Int. 2012;32:991–5.
13. Atwa MA, Balata MG, Hussein AM, Abdelrahman NI, Elminshawy HH. Serum 25-hydroxyvitamin D concentration in patients with psoriasis and rheumatoid arthritis and its association with disease activity and serum tumor necrosis factor-alpha. Saudi Med J. 2013;34:806–13.
14. Chen J, Liu W, Lin Q, Chen L, Yin J, Huang H. Vitamin D deficiency and low bone mineral density in native Chinese rheumatoid arthritis patients. Int J Rheum Dis. 2014;17:66–70.
15. Furuya T, Hosoi T, Tanaka E, Nakajima A, Taniguchi A, Momohara S, et al. Prevalence of and factors associated with vitamin D deficiency in 4,793 Japanese patients with rheumatoid arthritis. Clin Rheumatol. 2013;32:1081–7.
16. Haga H-J, Schmedes A, Naderi Y, Moreno AM, Peen E. Severe deficiency of 25-hydroxyvitamin D3 (25-OH-D3) is associated with high disease activity of rheumatoid arthritis. Clin Rheumatol. 2013;32:629–33.
17. Higgins MK, Mackie SL, Thalayasingam N, Bingham SJ, Hamilton J, Kelly CA. The effect of vitamin D levels on the assessment of disease activity in rheumatoid arthritis. Clin Rheumatol. 2013;32(6):863–7.
18. Sabbagh Z, Markland J, Vatanparast H. Vitamin D Status Is Associated with Disease Activity among Rheumatology Outpatients. Nutrients. 2013;5:2268–75.
19. Yazmalar L, Ediz L, Alpayci M, Hiz O, Toprak M, Tekeoglu I. Seasonal disease activity and serum vitamin D levels in rheumatoid arthritis, ankylosing spondylitis and osteoarthritis. Afr Health Sci. Makerere University Medical School; 2013;13(1):47–55.
20. Cote J, Berger A, Kirchner LH, Bili A. Low vitamin D level is not associated with increased incidence of rheumatoid arthritis. Rheumatol Int. 2014;34:1475–9.
21. Gheita TA, Sayed S, Gheita HA, Kenawy SA. Vitamin D status in rheumatoid arthritis patients: relation to clinical manifestations, disease activity, quality of life and fibromyalgia syndrome. Int J Rheum Dis. 2016;19:294–9.
22. Hong Q, Xu J, Xu S, Lian L, Zhang M, Ding C. Associations between serum 25-hydroxyvitamin D and disease activity, inflammatory cytokines and bone loss in patients with rheumatoid arthritis. Rheumatology. 2014;53:1994–2001.
23. Hiraki LT, Arkema E V., Cui J, Malspeis S, Costenbader KH, Karlson EW. Circulating 25-hydroxyvitamin D level and risk of developing rheumatoid arthritis. Rheumatology. 2014;53:2243–8.
24. Sahebari M, Mirfeizi Z, Rezaieyazdi Z, Rafatpanah H, Goshyeshi L. 25(OH) vitamin D serum values and rheumatoid arthritis disease activity (DA S28 ESR). Casp J Intern Med. 2014;5:148–55.
25. Sharma R, Saigal R, Goyal L, Mital P, Yadav RN, Meena PD, et al. Estimation of vitamin D levels in rheumatoid arthritis patients and its correlation with the disease activity. J Assoc Physicians India. 2014;62:678–81.
26. Cooles FA, Pratt AG, Lendrem DW, Ng WF, Aspray TJ, Isaacs JD. [Retrospective analysis of the role of serum vitamin D in early rheumatic disease.](https://www.ncbi.nlm.nih.gov/pubmed/25406355) Rheumatology (Oxford). 2015;54:374-5.
27. Raczkiewicz A, Kisiel B, Kulig M, Tłustochowicz W. Vitamin D status and its association with quality of life, physical activity, and disease activity in rheumatoid arthritis patients. J Clin Rheumatol. 2015;21:126–30.
28. Matsumoto Y, Sugioka Y, Tada M, Okano T, Mamoto K, Inui K, et al. Relationships between serum 25-hydroxycalciferol, vitamin D intake and disease activity in patients with rheumatoid arthritis –TOMORROW study. Mod Rheumatol. 2015 Mar 4;25:246–50.
29. Azzeh FS, Kensara OA. Vitamin D Is a Good Marker for Disease Activity of Rheumatoid Arthritis Disease. Dis Markers . 2015;2015:1–6.
30. Brance ML, Brun LR, Lioi S, Sánchez A, Abdala M, Oliveri B. Vitamin D levels and bone mass in rheumatoid arthritis. Rheumatol Int. 2015;35:499–505.
31. Cen X, Liu Y, Yin G, Yang M, Xie Q. Association between Serum 25-Hydroxyvitamin D Level and Rheumatoid Arthritis. Biomed Res Int. 2015;2015:913804.
32. Wang Y, ZHANG F, WANG S, SHANG X, LUO S, ZHOU H, et al. Serum Vitamin D Level is Inversely Associated With Anti-Cyclic Citrullinated Peptide Antibody Level and Disease Activity in Rheumatoid Arthritis Patients. Arch Rheumatol. Turkish League Against Rheumatism; 2016;31(1):64–70.
33. Cecchetti S, Tatar Z, Galan P, Pereira B, Lambert C, Mouterde G, et al. Prevalence of vitamin D deficiency in rheumatoid arthritis and association with disease activity and cardiovascular risk factors: data from the COMEDRA study. Clin Exp Rheumatol [Internet]. [cited 2018 Oct 14];34(6):984–90. Available from: http://www.ncbi.nlm.nih.gov/pubmed/27749232
34. Pakchotanon R, Chaiamnuay S, Narongroeknawin P, Asavatanabodee P. The association between serum vitamin D Level and disease activity in Thai rheumatoid arthritis patients. Int J Rheum Dis. 2016;19:355–61.
35. Zakeri Z, Sandoughi M, Mashhadi MA, Raeesi V, Shahbakhsh S. Serum vitamin D level and disease activity in patients with recent onset rheumatoid arthritis. Int J Rheum Dis. 2016 Apr;19:343–7.
36. Mateen S, Moin S, Shahzad S, Khan AQ. Level of inflammatory cytokines in rheumatoid arthritis patients: Correlation with 25-hydroxy vitamin D and reactive oxygen species. PLoS One. 2017;12:e0178879.
37. Hajjaj-Hassouni N, Mawani N, Allali F, Rkain H, Hassouni K, Hmamouchi I, et al. Evaluation of Vitamin D Status in Rheumatoid Arthritis and Its Association with Disease Activity across 15 Countries: “the COMORA Study.” Int J Rheumatol. 2017; 2017:5491676.
38. Vojinovic J, Tincani A, Sulli A, Soldano S, Andreoli L, Dall’Ara F, et al. European multicentre pilot survey to assess vitamin D status in rheumatoid arthritis patients and early development of a new Patient Reported Outcome questionnaire (D-PRO). Autoimmun Rev. 2017;16:548-554.
39. Herly M, Stengaard-Pedersen K, Vestergaard P, Østergaard M, Junker P, Hetland ML, et al. The D-vitamin metabolite 1,25(OH)2 D in serum is associated with disease activity and Anti-Citrullinated Protein Antibodies in active and treatment naïve, early Rheumatoid Arthritis Patients. Scand J Immunol. 2018;88:e12704.
40. de la Torre Lossa P, Moreno Álvarez M, González Guzmán MDC, López Martínez R, Ríos Acosta C. Vitamin D is not useful as a biomarker for disease activity in rheumatoid arthritis. Reumatol Clin. 2018; pii: S1699-258X(18)30077-9.
41. Khoja S, El-Miedany Y, Iyer A, Bahlas S, Balamash K, Elshal M. Associations of Vitamin D Levels and Vitamin D Receptor Genotypes with Patient-Reported Outcome/Disease Activity in Patients with Rheumatoid Arthritis. Clin Lab. 2018;64(01+02/2018).

**Supplementary Table 2. Vitamin D supplementation trials in rheumatoid arthritis**

| **Study** | **Study participants (no. eligible + DMARD tx)** | **Treatment groups / trial design/ BL vitD** | **Primary and secondary outcome measures** | **Summary of key findings** |
| --- | --- | --- | --- | --- |
| Andjelkovic et al. 1999 | RA=19 (on MTX +/- GC, active dx) | 2 microg/day oral alphacalcidiol for 3/12 in 2 groups; mod + highly active RA. Control group = same patients data collected over 3 months prior to suppl.  Open-label trial | ESR, CRP, EMS, Richie index, Lee index at 3 months | CRP, SJC, TJC, Richie index and Lee index all significantly decreased after 3/12.  RF and CRP were decreased, but this was not statistically significant. |
| Gopinath et al. 2011 | RA=121 (on triple DMARDs) | 500 IU 1,25OH2D3 + CaCO3 vs. CaCO3  Open-label  25OHD3 <20ng/ml at BL | Pain relief assessed by patient VAS at first relief of pain and again at 3/12 | No difference in time achieve first pain relief however there was higher pain relief in the vitD group at 3/12 (NNT=5) |
| Salesi et al. 2012 | RA=117 (on MTX +/- HCQ/ CQ, active dx) | 50,000 IU/ week for 3 months vs. placebo  Double blinded trial | >0.6 or >1.2 improvement in DAS28 at wk 12 | No improvement in outcome measures reported |
| Dehghan et al 2014 | RA=80 (remission for 2/12) | Choleclaciferol 50,000IU/week vs. placebo  Double-blind RCT.  25OHD levels were <30ng/ml at BL | DAS28 as a marker of relapse, over 6/12 | No statistical significant reduction in relapse rate was observed |
| Yang J et al, 2015 | RA=377 (RA in remission) | Alfacalcidol 0.25 microg BD for 24 months in vitD def. RA vs. placebo vs. RA with normal vit D levels and no treatment  Open-label  Deficiency = 25OHD3 <30ng/ml | VAS, SJC, TJC, CRP, ESR and DAS-28 every 2-3/12 | Normal vit D assoc. with ↓recurrence. No difference was observed with or without vit D suppl. In RA with low vitD |
| Buondonno et al. 2017 | Early RA=39 (Tx naïve), HC=31 | MTX + GC vs. MTX + GC + 300,000 IU (one-off dose)  Double-blind RCT | T cell phenotypes, OC precursors, inflammatory cytokines, clinical parameters at 3/12 | Reduced IL-23, incr. GHS reported in the vitD suppl. group |
| Chandrashekara et al. 2017 | RA = 73 (on DMARDs, active dx) | 60, 000 IU/ week for 6 weeks then 60,000 IU/month for 3/12  Open-label  25OHD3 <20ng/ml at BL + DAS28-CRP >2.6 | Improvement in DAS28-CRP, vitamin D status | ↓ DAS28-CRP and ↑ vitD >20ng/ml in the tx group |

BD: twice daily; BL: baseline; CQ: chloroquine; CRP: C-reactive protein; DAS: disease activity score; DMARDs: disease-modifying anti-rheumatic drugs; EMS: early morning stiffness; ESR: erythrocyte sedimentation rate; GC: glucocorticoids; HCQ: hydroxychloroquine; MTX: methotrexate; NNT: number needed to treat; RA: rheumatoid arthritis; RF: rheumatoid factor; SJC: swollen joint count; TJC: tender joint count; Tx: treatment; VAS: visual analogue scale.

**Reference list**

1. Andjelkovic Z, Vojinovic J, Pejnovic N, Popovic M, Dujic A, Mitrovic D, et al. Disease modifying and immunomodulatory effects of high dose 1 alpha (OH) D3 in rheumatoid arthritis patients. Clin Exp Rheumatol. 1999;17:453–6.
2. Gopinath K, Danda D. Supplementation of 1,25 dihydroxy vitamin D3 in patients with treatment naive early rheumatoid arthritis: a randomised controlled trial. Int J Rheum Dis [Internet]. 2011;14:332–9.
3. Salesi M, Farajzadegan Z. Efficacy of vitamin D in patients with active rheumatoid arthritis receiving methotrexate therapy. Rheumatol Int. 2012;32:2129–33.
4. Dehghan A, Rahimpour S, Soleymani-Salehabadi H, Owlia MB. Role of vitamin D in flare ups of rheumatoid arthritis. Z Rheumatol [Internet]. 2014;73:461–4.
5. Yang J, Liu LIN, Zhang Q, Li M, Wang J. Effect of vitamin D on the recurrence rate of rheumatoid arthritis. 2015;1812–6.
6. Buondonno I, Rovera G, Sassi F, Rigoni MM, Lomater C, Parisi S, et al. Vitamin D and immunomodulation in early rheumatoid arthritis : A randomized double- blind placebo-controlled study. PLoS One. 2017;12(6):e0178463.
7. Chandrashekara S, Patted A. Role of vitamin D supplementation in improving disease activity in rheumatoid arthritis: An exploratory study. Int J Rheum Dis. 2017;20:825–31.
